# The complete mitochondrial genome of *Lamprologus signatus* (Perciformes: Cichlidae)

**DOI:** 10.1080/23802359.2021.1981789

**Published:** 2021-11-28

**Authors:** Sang-Eun Nam, Hye-Jin Eom, Hyoung Sook Park, Jae-Sung Rhee

**Affiliations:** a Department of Marine Science, College of Natural Sciences, Incheon National University, Incheon, South Korea; b Department of Song-Do Bio-Environmental Engineering, Incheon Jaeneung University, Incheon, South Korea; c Research Institute of Basic Sciences, Incheon National University, Incheon, South Korea; d Institute of Green Environmental Research Center, Incheon, South Korea

**Keywords:** Complete mitogenome, cichlid fish, *Lamprologus signatus*, phylogenetic analysis

## Abstract

In this study, the complete 16,583 bp mitochondrial genome of *Lamprologus signatus* (Poll, 1952) was determined from a specimen sourced from Lake Tanganyika. The mitogenome contains 37 genes [13 protein-coding genes (PCGs), two ribosomal RNA (rRNA) genes, and 22 transfer RNA (tRNA) genes] and a putative control region, which consists of 27.1% A, 27.0% T, 29.9% C, and 16.0% G, with a total G + C content of 45.9%. A maximum likelihood phylogenetic tree based on mitochondrial PCGs suggested that *L. signatus* is clustered with members of the tribes Haplochromini and Tropheini. As this is the first report of the entire mitogenome in the tribe Lamprologini, the complete mitochondrial sequence information of *L. sigantus* will be useful in determining phylogenetic relationships of Pseudocrenilabrinae tribes.

Cichlids are one of the most species-rich acanthomorphs (spiny-rayed fish) with numerous species (>1700 species described), different evolutionary lineages, large genetic diversity, and significant ecological and morphological divergences (Kornfield and Smith 2000; Nelson et al. 2016). They are widely distributed from Africa to South America and Middle America (Smith et al. 2008). The predominantly biodiverse and adaptive radiations of cichlids have been observed in three Great Lakes of East Africa, including Lake Tanganyika, Lake Victoria, and Lake Malawi. Of these, Lake Tanganyika is a deep tropical and large Rift Valley lake with an age of 9–12 million years (Irisarri et al. 2018) and is ecologically important for the study of different biomes, including pelagic, benthic, and littoral ecosystems (Iliffe 1979). Lake Tanganyika too, is an invaluable region for studying adaptive diversification and colonization scenarios of fish, particularly for cichlids, which represent the major species-rich vertebrate radiations (Muschick et al. 2012; Brawand et al. 2014). Cichlids in Lake Tanganyika still show rapid diversification, as revealed by hybridization events occurring in two colonizing lineages at the onset of the radiation (Irisarri et al. 2018). The cichlid fauna of Lake Tanganyika has its roots in one subgroup of the monophyletic African cichlid assemblage (Schwarzer et al. 2009) and has been divided into 14 taxonomic subgroups, referred to as ‘tribes’ (Dunz and Schliewen 2013). *Lamprologus signatus* (Poll, 1952) is a species of the non-mouthbrooding cichlid tribe Lamprologini that is endemic to Lake Tanganyika. Due to ease of handling and maintenance in aquariums, *L. signatus* has great economic value as an ornamental species in the aquatic trade industry. The origin and distribution of Lamprologini is controversial. Almost all Lake Tanganyika tribes are endemic to the Great Lakes; however, members of the tribe Lamprologini are distributed in rivers across East and Central Africa (Schelly and Stiassny 2004). Several studies suggest that Lamprologini solely evolved as individual radiation in Lake Tanganyika and further contributed to the Congo Basin colony (Salzburger et al. 2002; Day et al. 2007; Sturmbauer et al. 2010), whereas the Congo Lamprologini colony was suggested to be a relict ancestral species of the Lake Tanganyika Lamprologini radiation (Clabaut et al. 2005). Thus, comprehensive mitogenomic information of *L. signatus* would be advantageous in establishing the precise phylogenetic placement of Lamprologini and understanding its Lake Tanganyika colonization scenarios.

There is no information on the complete mitogenome in the tribe Lamprologini, although their incomplete mitochondrial genome and genomic markers have been registered in the NCBI GenBank database. Several specimens of *L. signatus* were collected at Wonzye Point (8°43′ N 31°07′ E), Zambia, which were procured through aquarium trade. The specimens and DNA were deposited in the fish collection at the Research Institute of Basic Sciences of Incheon National University (Specimen ID: 2013-Cichlidae-13; https://www.inu.ac.kr/user/indexMain.do?siteId=ribs; Dr. Sang-Eun Nam; se_nam2@inu.ac.kr). Total genomic DNA was prepared from a specimen muscle using a DNeasy Blood and Tissue kit (Qiagen, Hilden, Germany) according to the manufacturer’s standard protocol. Since next-generation sequencing (NGS) technology has been widely applied for the assembly of complete mitogenomes in fish, the Illumina NGS platform was used in this study. A fragment library was prepared with the total genomic DNA using the TruSeq DNA Sample Preparation Kit (Illumina, San Diego, CA, USA) as previously described (Nam and Rhee 2020), before sequencing using an Illumina HiSeq sequencer. The sequencing library was prepared by random fragmentation of the DNA sample (<600 bp), followed by 5′ and 3′ adapter ligation. Raw reads were obtained from the sample that passed the quality control check on the Illumina HiSeq platform (Illumina) at Macrogen, Inc. (Seoul, South Korea). Adapter sequences, low quality reads, reads with >10% of unknown bases, and ambiguous bases were removed to obtain high quality assembly by using Trimmomatic (Bolger et al. 2014). After the quality check process, 36,998,926 filtered reads were obtained from 46,566,952 raw reads. Subsequently, *de novo* assembly was conducted with various k-mers using SPAdes (Bankevich et al. 2012), and a circular contig of the *L. signatus* mitogenome was obtained. The resulting contig consensus sequence was annotated using MITOS2 (Bernt et al. 2013) and tRNAscan-SE 2.0 (Lowe and Eddy 1997). BLAST searches confirmed the identity of the genes (http://blast.ncbi.nlm.nih.gov).

The complete mitochondrial genome of *L. signatus* was 16,583 bp in length (GenBank accession no. MZ427900) and contained 13 protein-coding genes (PCGs), two ribosomal RNA (rRNA) genes (12S and 16S), and 22 transfer RNA (tRNA) genes. For the 13 PCGs, the most common shared start codon was ATG (all PCGs except for *cox1*), followed by GTG (*cox1*). The most common termination codon was TAA (*atp8*, *atp6*, *cox3*, *nad4l*, *nad5*, *nad6*, *cob*, *nad1*, and *nad2*). The gene order and composition of the *L. signatus* mitogenome were identical to those of other cichlid mitogenomes.

To determine the phylogenetic relationship of *L. signatus*, a concatenated dataset of the 13 PCGs from the *L. signatus* mitogenome, closely related 31 published complete mitogenomes of cichlids, and an outgroup from the family Balistidae available from GenBank was used to build maximum-likelihood (ML) phylogeny. JModelTest ver. 2.1.10 (Darriba et al. 2012) was used to select the best substitution model, and a substitution model (HKY + G + I) was applied to perform an ML method in PhyML 2.4.5 (Guindon and Gascuel 2003) with 1000 bootstrap replicates. The overall topology of each tribe was consistent with previous phylogenetic results (Schwarzer et al. 2009; Irisarri et al. 2018) (Figure 1). The *L. signatus* mitogenome, a member of Lamprologini, was placed as a sister lineage to the members of Haplochromini (including Tropheini), as established in previous studies (Schwarzer et al. 2009; Meyer et al. 2015; Irisarri et al. 2018).

**Figure 1.**
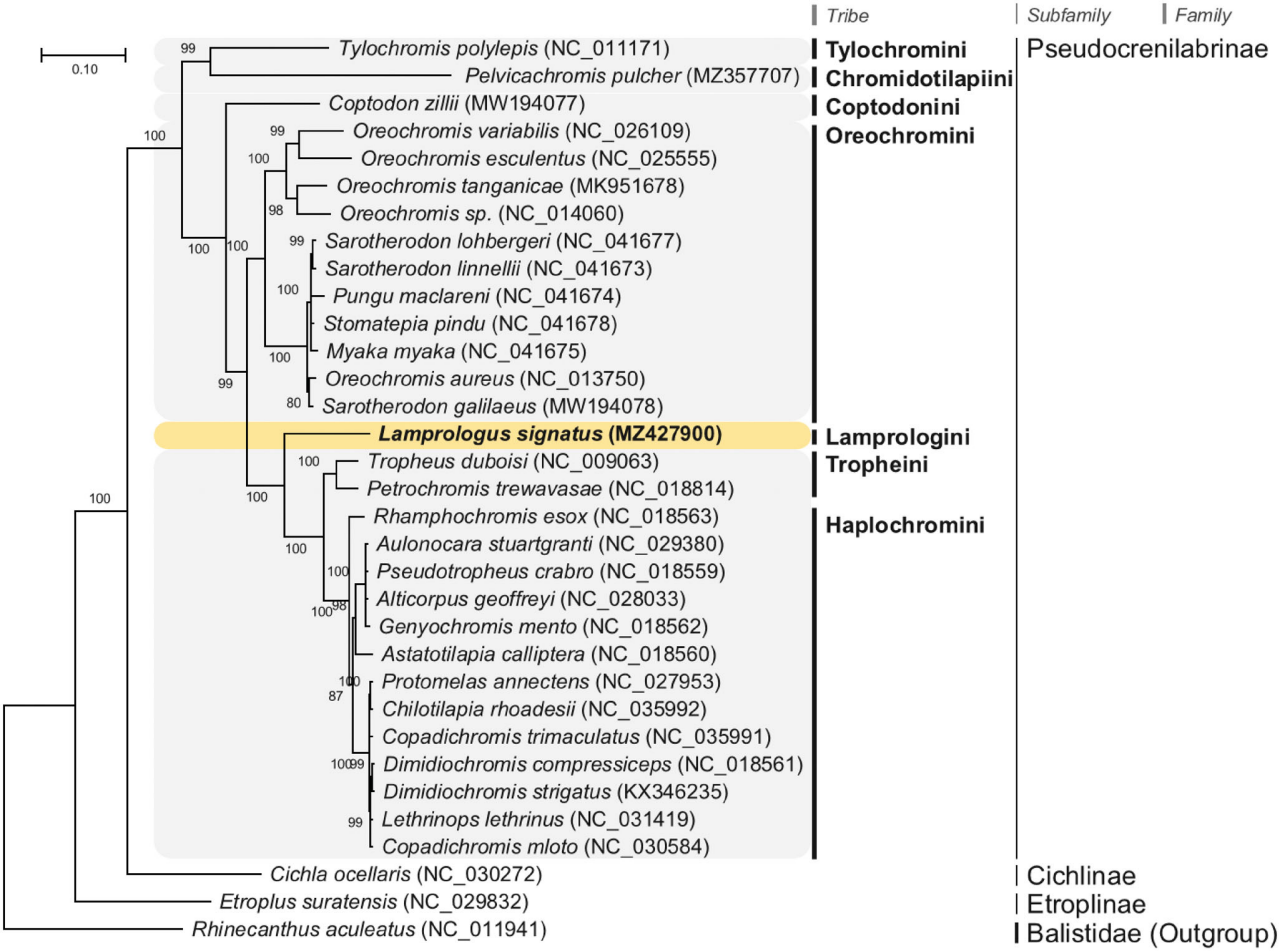
Maximum-likelihood (ML) phylogeny of 31 published complete mitogenomes of cichlids and an outgroup from the family Balistidae based on the concatenated nucleotide sequences of protein-coding genes (PCGs). The phylogenetic analysis was performed using the maximum likelihood method, GTR + G + I model with a bootstrap of 1000 replicates. Numbers on the branches indicate ML bootstrap percentages. DDBJ/EMBL/Genbank accession numbers for published sequences are incorporated. The black triangle indicates the cichlid analyzed in this study.

## Data Availability

BioProject, BioSample, and SRA accession numbers are https://www.ncbi.nlm.nih.gov/search/all/?term=PRJNA550295, https://www.ncbi.nlm.nih.gov/biosample/SAMN19803888, and https://www.ncbi.nlm.nih.gov/sra/?term=SRR15356509, respectively. The data that support the findings of this study are openly available in the National Center for Biotechnology Information (NCBI) at https://www.ncbi.nlm.nih.gov, with an accession number MZ427900.
